# RIPK3-dependent cell death and inflammasome activation in FLT3-ITD expressing LICs

**DOI:** 10.18632/oncotarget.11195

**Published:** 2016-09-10

**Authors:** Ulrike Höckendorf, Monica Yabal, Philipp J. Jost

**Affiliations:** III. Medizinische Klinik für Hämatologie und Internistische Onkologie, Klinikum rechts der Isar, Technische Universität München, Munich, Germany

**Keywords:** AML, RIPK3, regulated necrosis, leukemia initiating cells, inflammasome

Acute myeloid leukemia (AML) is sustained by leukemia-initiating cells (LICs) with blocked myeloid differentiation and increased self-renewal capacities. These cells arise from pre-leukemic hematopoietic stem and progenitor cells (HSPCs) that carry genetic alterations being selected for during leukemogenesis [1]. The resistance of LICs to standard chemotherapies presents a major clinical challenge as they eventually cause disease relapse and death [2].

Understanding the mechanisms of LIC resistance to undergoing cell death is therefore critical for a curative therapy of AML. The ability of LICs and pre-leukemic HSCs to evade cell death has been attributed to several pathways that are distinct from normal HSPCs. LICs for example have constitutive NF-κB activity while normal HSPCs do not. In addition, analysis of patient-derived cells showed that in some cases the apoptotic cell death pathway in LICs is skewed.

However, work in mature myeloid cells has shown that these cells are exquisitely sensitive to undergoing cell death driven by receptor-interacting serine-threonine kinase 3 (RIPK3) [3]. In addition to inducing apoptosis, RIPK3 can also induce regulated necrosis. In contrast to apoptosis, regulated necrosis in myeloid cells is accompanied by inflammation due to the release of cytokines and DAMPs from dying cells. We found that in a similar fashion LICs are also sensitive to RIPK3-dependent cell death and that loss of *RIPK3* expression underlies the developmental block and oncogenic process in many subtypes of AML [4].

In our study, we focused on one of the recurring genetic alterations found in AML that gives rise to internal tandem duplications of the tyrosine kinase receptor FLT3 (FLT3-ITD). In human patients FLT3-ITD is associated with poor prognosis and lower overall survival. Using a model of adoptive transfer of bone marrow (BM) cells transduced with FLT3-ITD we demonstrated that in the absence of *Ripk3,* FLT3-ITD-induced leukemia was accelerated and significantly more aggressive. The enhanced disease was due to the expansion of common myeloid progenitors (CMPs) and short-term hematopoietic stem cells (ST-HSCs), being the cellular compartment to contain LICs. RIPK3 functioned to induce cell death of these cells and acted as a tumor suppressor. We further showed that RIPK1 and the TNF receptors 1 and 2 (TNFR1/2) functioned upstream of RIPK3 to regulate cell death. However, *Mlkl* deficiency did not abrogate cell death *in vitro*, and disease progression *in vivo* was also not significantly different, suggesting that RIPK3 may also induce apoptosis of LICs, a function that is consistent with recently published results [5].

**Figure 1:**
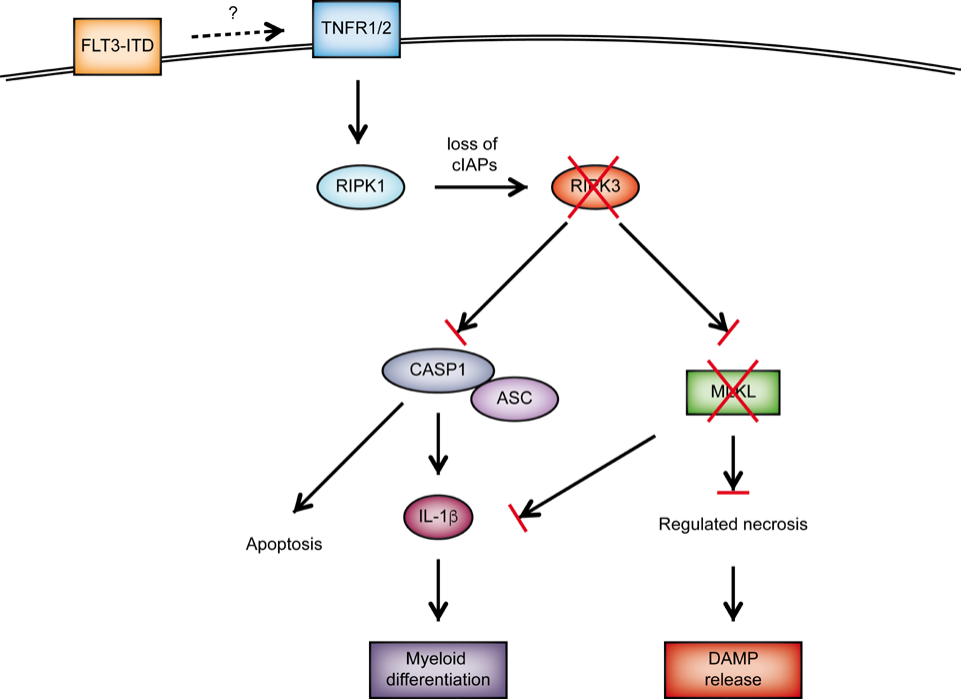
Schematic diagram of RIPK3-dependent cell death and inflammasome activation in FLT3-ITD expressing LICs. Mutated FLT3 signaling in transformed LICs propagates proinflammatory signaling, which results in substantial cytokine secretion. Subsequent activation of TNF receptors 1 and 2 activates RIPK1/RIPK3, together with loss of cIAPs resulting in the induction of regulated necrosis and inflammasome formation. Several AML subtypes including FLT3-ITD+ AML show reduced expression of RIPK3 and MLKL, preventing LIC cell death and IL-1β production. Thus, regulated necrosis serves as a tumor suppressive mechanism by inducing cell death of LICs as well as by promoting myeloid differentiation of leukemic stem/progenitor cells by IL-1β signaling.

Importantly, WT FLT3-ITD-transformed cells produced substantial amounts of the inflammatory cytokine IL-1β which was severely blunted by *Ripk3* or *Mlkl* deficiency. The role of IL-1β in promoting LIC differentiation and thus restricting leukemogenesis was confirmed with *Pycard^−^* and *Il1r1^−^* HSPCs. Hence, RIPK3 signals through two complementary pathways, cell death and IL-1β production, to restrict LICs and to promote myeloid cell differentiation, respectively (Figure 1).

Using several human patient sample cohorts, we observed a reduced expression of both *RIPK3* and *MLKL* in FLT3-ITD-associated AML, and many other AML subtypes. The reduction of *RIPK3* expression was specific to AML as we did not observe changes in *RIPK3* expression in patient cohorts diagnosed with other myeloid proliferative neoplasms. These findings supported our hypothesis that RIPK3 acts as a tumor suppressor in AML.

Exceptions within the AML cohorts were patients harboring mixed-lineage leukemia (MLL) translocations which showed normal *RIPK3* and *MLKL* expression. In a mouse model we confirmed that *Ripk3* deficiency did not alter the myeloproliferative neoplasm caused by MLL-ENL. These finding are consistent with independent studies showing that deletion of *Ripk3* did not alter disease progression of MLL-ENL-, MLL-AF9-, NUP98-HoxA9-, and HoxA9/Meis1-induced leukemia [6, 7]. Furthermore, the authors showed that these leukemia subsets were sensitive to killing by the SMAC mimetic birinapant in a RIPK1/TNFR1-dependent manner. Therefore, our work suggests that alternative therapeutic approaches will be required for AML subsets with reduced *RIPK3* expression.

Hence, RIPK3 and MLKL have pivotal roles in a broad, but not all-inclusive, range of AML subtypes. Loss of this signaling pathway acts as a double-edged sword; it first promotes survival of transforming HSPCs and secondly inhibits LIC differentiation along the myeloid lineage. Together, this leads to the accumulation of LICs and thus leukemogenesis. The finding that RIPK3 promotes inflammatory cell death and differentiation of LICs supports a role for RIPK3 as an AML tumor suppressor, and suggests reactivation of RIPK3 as a potential therapeutic approach in some AML subtypes.
